# Diffusion Model Based Spectral Clustering for Protein-Protein Interaction Networks

**DOI:** 10.1371/journal.pone.0012623

**Published:** 2010-09-07

**Authors:** Kentaro Inoue, Weijiang Li, Hiroyuki Kurata

**Affiliations:** 1 Department of Bioscience and Bioinformatics, Kyushu Institute of Technology, Iizuka, Japan; 2 The Key Laboratory of Industrial Biotechnology, Southern Yangtze University, Wuxi, China; Karolinska Institutet, Sweden

## Abstract

**Background:**

A goal of systems biology is to analyze large-scale molecular networks including gene expressions and protein-protein interactions, revealing the relationships between network structures and their biological functions. Dividing a protein-protein interaction (PPI) network into naturally grouped parts is an essential way to investigate the relationship between topology of networks and their functions. However, clear modular decomposition is often hard due to the heterogeneous or scale-free properties of PPI networks.

**Methodology/Principal Findings:**

To address this problem, we propose a diffusion model-based spectral clustering algorithm, which analytically solves the cluster structure of PPI networks as a problem of random walks in the diffusion process in them. To cope with the heterogeneity of the networks, the power factor is introduced to adjust the diffusion matrix by weighting the transition (adjacency) matrix according to a node degree matrix. This algorithm is named adjustable diffusion matrix-based spectral clustering (ADMSC). To demonstrate the feasibility of ADMSC, we apply it to decomposition of a yeast PPI network, identifying biologically significant clusters with approximately equal size. Compared with other established algorithms, ADMSC facilitates clear and fast decomposition of PPI networks.

**Conclusions/Significance:**

ADMSC is proposed by introducing the power factor that adjusts the diffusion matrix to the heterogeneity of the PPI networks. ADMSC effectively partitions PPI networks into biologically significant clusters with almost equal sizes, while being very fast, robust and appealing simple.

## Introduction

A goal of systems biology is to analyze large-scale molecular networks including gene expressions and protein interactions, revealing the relationships between network structures and their biological functions. Generally it is not feasible to understand the whole networks as they are. A common way to network analysis is to partition the network into subnetworks responsible for specific biological functions. Since biological functions can be carried out by particular groups of genes and proteins, dividing networks into naturally grouped parts (clusters or communities) is an essential way to investigate some relationships between the function and topology of networks or to reveal hidden knowledge behind them. Especially, protein-protein interaction (PPI) networks attract biologists to understand the whole image of cellular systems.

PPI networks can generally be transformed into a graph, where a node is the molecule and an edge is the interaction. Large size and high heterogeneity are common features to PPI networks. A few nodes have very large degrees, while others have very few interactions. Classical graph-based agglomerative methods employ a variety of similarity measures between nodes to partition PPI networks, but they often result in a poor clustering arrangement that contains one or a few giant core clusters with many tiny ones [1]. To improve the clustering results, PPI networks were weighted based on topological properties such as shortest path length [2], [3], clustering coefficients [4], node degree, or the degree of experimental validity [5]. The problem, however, still remains to be solved.

As a new type of clustering algorithms, the edge-betweenness was defined as a global measure to separate PPI networks into subgraphs in a divisive manner [6]–[9]. Edge-betweenness is the number of shortest paths between all pairs of nodes that run through the edge. It is able to identify biologically significant modular structures, but it requires lots of computation resources. As an approach to coordination of typical clustering algorithms, an ensemble method was proposed to combine multiple, independent clustering arrangements to deduce a single consensus cluster structure [10].

Not only network partition but also extraction of protein complexes have been performed to analyze PPI networks. To detect such densely connected subgraphs in them, many algorithms were proposed. Molecular Complex Detection (MCODE) is based on node weighting by local neighborhood density and outward traversal from a locally dense seed protein to isolate densely connected regions [11]. The Restricted Neighborhood Search Clustering Algorithm (RNSC) is a cost based local search algorithm to explore the solution space to minimize cost function, calculated according to the numbers of intra-cluster and inter-cluster edges [12]. Spectral analysis was used to identify protein complexes by investigating the eigenvalues/eigenvectors of the matrices that express node connectivity [13], [14]. Physical model-based algorithms were presented, such as Markov Clustering (MCL) [15] and Superparamagnetic Clustering (SPC) [16], to identify densely connected regions. MCL is a fast and scalable unsupervised clustering algorithm for graphs, controlled by alternation of two operators: inflation and expansion. SPC is a hierarchical clustering algorithm inspired from an analogy with the physical properties of a ferromagnetic model subject to fluctuation at nonzero temperature. Usually, these algorithms may miss many peripheral proteins that connect to the core complex clusters with few links, even though these peripheral proteins represent true interactions experimentally verified.

Heuristic rule-based algorithms were proposed to reveal the structure of PPI networks [17], [18]. A layered clustering algorithm was presented, which groups proteins by the similarity of their direct neighborhoods to identify locally significant proteins that links different clusters, called mediators [19]. Power graph analysis transforms biological networks into a compact, less redundant representation, exploiting the abundance of cliques and bicliques as elementary topological motifs [20].

Those proposed algorithms were characterized in terms of many criteria: calculation speed, modularity, cluster size, and biological significance. On the other hand, interestingly, spectral clustering analysis, which is an appealing simple and theoretically sound method [20]–[24], has hardly been studied to partition PPI networks, while it is used for detecting protein complexes [25], [26].

In this study, to explore a biologically meaningful partition of PPI networks, we propose a new diffusion model-based spectral clustering method by introducing a power factor that adjusts the diffusion matrix to the heterogeneity of PPI networks. It is named the adjustable diffusion matrix-based spectral clustering (ADMSC). Discovering cluster structures by random walks on the diffusion model is attributed to the spectral graph theory that solves the eigenvectors of Laplacian matrix [27], [28].

## Methods

### ADMSC: Diffusion matrix-based spectral clustering

Spectral analysis is performed for clustering or low dimensional representation of high dimensional data, based on the eigenvectors of the graph Laplacian on the data. Spectral analysis can be interpreted as a diffusion based probabilistic model [27]–[30]. We consider the following diffusion process of a particle on an undirected graph network with *n* nodes: 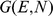. It can be fully described by its adjacency matrix 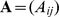 where 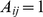 if there is an edge between nodes *i* and *j*; 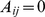 otherwise. The degree of node *i* is denoted by 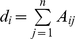, which is the number of connections of node *i*.

A particle randomly travels among the sites corresponding to nodes of the network. If the particle is at site *i* at time *t*, it will move to site *j* at time 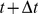 with probability 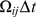. The matrix 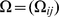, called the transition matrix, is defined according to the network topology. Denoting the probability distribution: 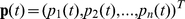, where 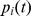 is the probability of finding the particle at site *i* at time *t*, we have the master equation:
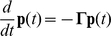
(1)where
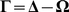
(2)is the diffusion matrix, and 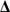 is a diagonal matrix given by:
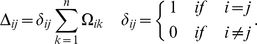
(3)


In this study, we propose the following form of transition matrix:
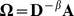
(4)where 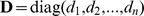 is the diagonal matrix of node degrees. A power factor of β is the adjustable parameter that critically controls clustering results. The introduction of β is the novel algorithm that enables clear decomposition of a scale-free network. Note that ADMSC (β = 1) corresponds to the regular spectral analysis, as illustrated in Table S1. The diffusion matrix can be symmetrized by the following transformation:
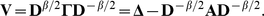
(5)Let:
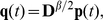
(6)Eq. (1) becomes:
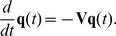
(7)Suppose the spectral decomposition of 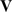 is:
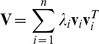
(8)where 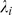 and 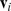 are the eigenvalues and the corresponding normalized eigenvectors of 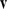, respectively. Since 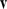 is symmetric, all eigenvalues 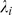 are real and the eigenvectors are orthogonal:
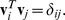
(9)Since 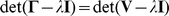, 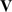 and 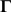 have exactly the same eigenvalues. It can be easily to verify that the eigenvectors of 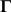 are 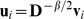 with corresponding eigenvalues 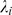. The eigenvectors are orthogonal on 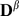,
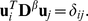
(10)


The solution of the diffusion equation (1) is:
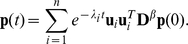
(11)Since 
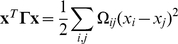
, 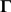 is positive semidefinite and thus all its eigenvalues are non-negative. Since each row of 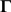 sums to 0, the smallest eigenvalue must be 0 and the associated eigenvector have the same value for its all components. Suppose the eigenvalues are sorted in ascending order:
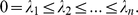
(12)


### Dynamics of particle diffusion

From Eq. (11) we see that when 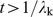 all the modes with eigenvalues larger than 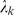 vanish quickly. So the eigenvalues are the vanishing speeds of the associated modes. In the process of diffusion, the time interval of two sequential modes *i* and *i*+1 is characterized by 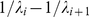. If a value of 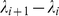 is small, the two modes disappear almost simultaneously. Therefore, a large gap in the eigenvalue profile is a characteristic sign of cluster structure. The larger the gap is, the clearer cluster structure there is. To reveal the cluster behavior, the slow modes are of interest. If the expansion of Eq. (11) is truncated at *i* = *k*, the result is called a *k*-slow-mode approximation. The network can be partitioned by analyzing the slow modes. The fast modes behave as noises that distort or blur the cluster structure. Notice that it is harmful to use more modes than just needed to reveal the best cluster structure.

### Geometric representation of diffusion map

Under the slow-mode approximation:
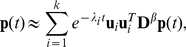
(13)to partition the network according to the *k* slow modes, we define a distance measure between the nodes. Consider the initial states that the walker starts at time 0 from a given site *k*, i.e., 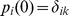. Denote the probability that the walker is at site *i* at time *t* by 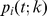, which can be viewed as the response of node *i* to a stimulus at node *k*. Define the diffusion distance between two nodes *i* and *j* as the weighted sum of the squared differences between the responses of all stimuli [28], [31]:
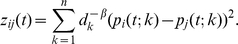
(14)It can be proved:
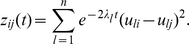
(15)When approximated with the slowest *k* modes, the discrepancy when *t*→0 is:
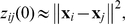
(16)where
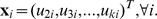
(17)Notice that the first eigenvector **u**_1_ is a constant vector, so it does not contribute to the distance. {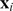} of Eq. (17), which is denoted the diffusion map, can be treated as a geometric representation of the network in a (*k*-1)-dimensional space in which the distance between the representative points is a measure of correlation between the nodes.

### Clustering of a diffusion map

With the help of the geometric representation (Eq. (17)), clustering can be performed by the k-means algorithm or the complete linkage method [28], [31]. As a quantitative measure of the similarity between a pair of nodes, angular distance is employed, which is defined as the angle between the vectors joining the origin of the (*k*-1)-dimensional space with the two points under consideration [32]. This distance is the key metrics to identify cluster structures in the diffusion map. Other distances, such as Euclidean distance and Manhattan distance, lead to generation of giant clusters. All calculations are performed by Matlab (Table S2, ADMSC.zip). The cluster number is determined to maximize the modularity in the same manner as the previous work [33].

### Measures for clustering performance

The network partition problem is in general defined as the division of a network into groups of approximately equal sizes, minimizing the number of edges between groups. Neither tiny clusters with one or a few nodes nor a dominant giant cluster are preferable for network partition. Furthermore, biologically significant functions should be assigned to each cluster.

#### Basic measures of identified clusters

To characterize the basic properties of the clusters generated by ADMSC, the cluster number, cluster size, and coefficient of variation (CV) of cluster size are calculated. When *k*-1 eigenvectors are employed as the diffusion map, the cluster number is set to *k*. The cluster size indicates the number of protein nodes within each cluster. The CV of cluster size is calculated over all the clusters. A large value of CV indicates the cluster size is greatly different; a low value of it shows they are almost the same.

#### Modularity

The modularity, originally proposed by Newman and Girvan, is employed to measure the topology-based modular property [9]. The modularity is defined by:
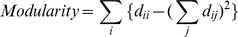
(18)It uses a 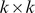 symmetric matrix of clusters. Each element 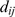 represents the fraction of the edges that link nodes between clusters *i* and *j*; each 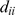 presents the fraction of the edges linking nodes within cluster *i*.

#### Cluster mapping score

We test if the clusters estimated correspond to functional annotations deriving from the Gene Ontology (GO) Consortium Online Database (http://www.geneontology.org/). Two vocabularies: cellular component (CC) and biological process (BP), are used to annotate proteins within clusters [34] (http://go.princeton.edu/cgi-bin/GOTermFinder). We remove GO annotations with evidence codes: IEA, RCA, IPI and ND, and exclude the BP and CC terms that annotate more than 100 proteins or fewer than three proteins.

Two measures for evaluating clusters: JaccardC measure and PRC measure, are used that are based on overlaps between the estimated clusters and the known groups of proteins with functional annotations [35]. Each measure gives a value in the range of 0 to 1, where higher values correspond to better overlaps. Here, let *M* be the number of clusters given by a particular clustering and *N* is the number of groups with respect to which we evaluate. The Jaccard similarity coefficient is provided as the size of the intersection over the size of the union. For sets of proteins corresponding to cluster *j* and group *i*, as follows:
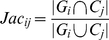
(19)where 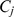 is the set of proteins within cluster *j* and 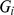 is the set of proteins associated with group *i*. The precision-recall (PR)-based score is presented for the sets of proteins corresponding to cluster *j* and group *i* by:
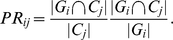
(20)The first (precision) part 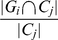 measures what fraction of the proteins in the cluster corresponds to the groups. The second (recall) part measures how much of group *i* is recovered by cluster *j*.

Before calculating the scores for mapping the estimated clusters on the known functional module groups, we consider the unclustered proteins as singleton clusters, remove all proteins in the functional groups that are not included in the network of interest. The mapping scores for clustering are defined that measure how well clusters map to known groupings of proteins. For each cluster 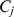, we find the group 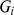 that maximizes the overlap between it and cluster 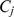, to present:
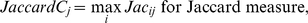
(21)

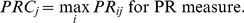
(22)For a singleton cluster 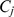, 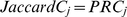 are set to zero. For each measure, an average over the clusters, weighted by cluster size, are used to calculate JaccardC and PRC:
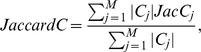
(23)

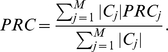
(24)


### Robustness analysis

Generally PPI data contain experimental errors that cannot be neglected, false positive and false negative data. The error rate depends strongly on employed high-throughput methods. To evaluate the robustness of ADMSC with respect to such data errors, we randomly perturbed the network topology by replacement of edges.

### Reference clustering algorithms

As reference algorithms, Markov clustering (MCL) and the shortest path betweenness (SPB) method are employed. MCL simulates random walks within a graph by alternation of two operators called inflation and expansion [15]. The MCL algorithm was compared with other methods RNSC [12], SPC [16], [25], and MCODE [11] by characterizing the resultant clusters with known annotated complexes [36]. MCL outperformed the other methods in terms of the correct identification of biologically significant complexes. Thus, MCL is used for our comparison. SPB is a divisive algorithm where edge-betweenness is defined as the global measure that is the number of shortest paths between all pairs of nodes that run through the edge [8], [9]. Edges between modules tend to have more shortest paths running through them than edges inside modules, thus show higher betweenness values. The deletion of edges with high betweenness can separate the network, while keeping the modular structure intact.

### Dataset

A PPI network of *Saccharomyces cerevisiae* is used as a model [37], which has 4902 nodes (proteins) and 17246 edges. This network shows a typical scale-free degree distribution (Figure S1), where the average values of the node degree and clustering coefficient are 7.04 and 0.126, respectively. In addition, the PPI networks of *Escherichia coli* and *Caenorhabditis elegans* are employed [37]. The former has 1447 nodes with 5879 edges, a cluster coefficient of 0.195 and an average degree of 8.13; the latter has 2385 nodes with 3825 edges, a cluster coefficient of 0.126 and an average degree of 3.21. For checking network maps, they are layouted or visualized by CADLIVE [38]–[40].

## Results

### Cluster number determination

**Figure 1:**
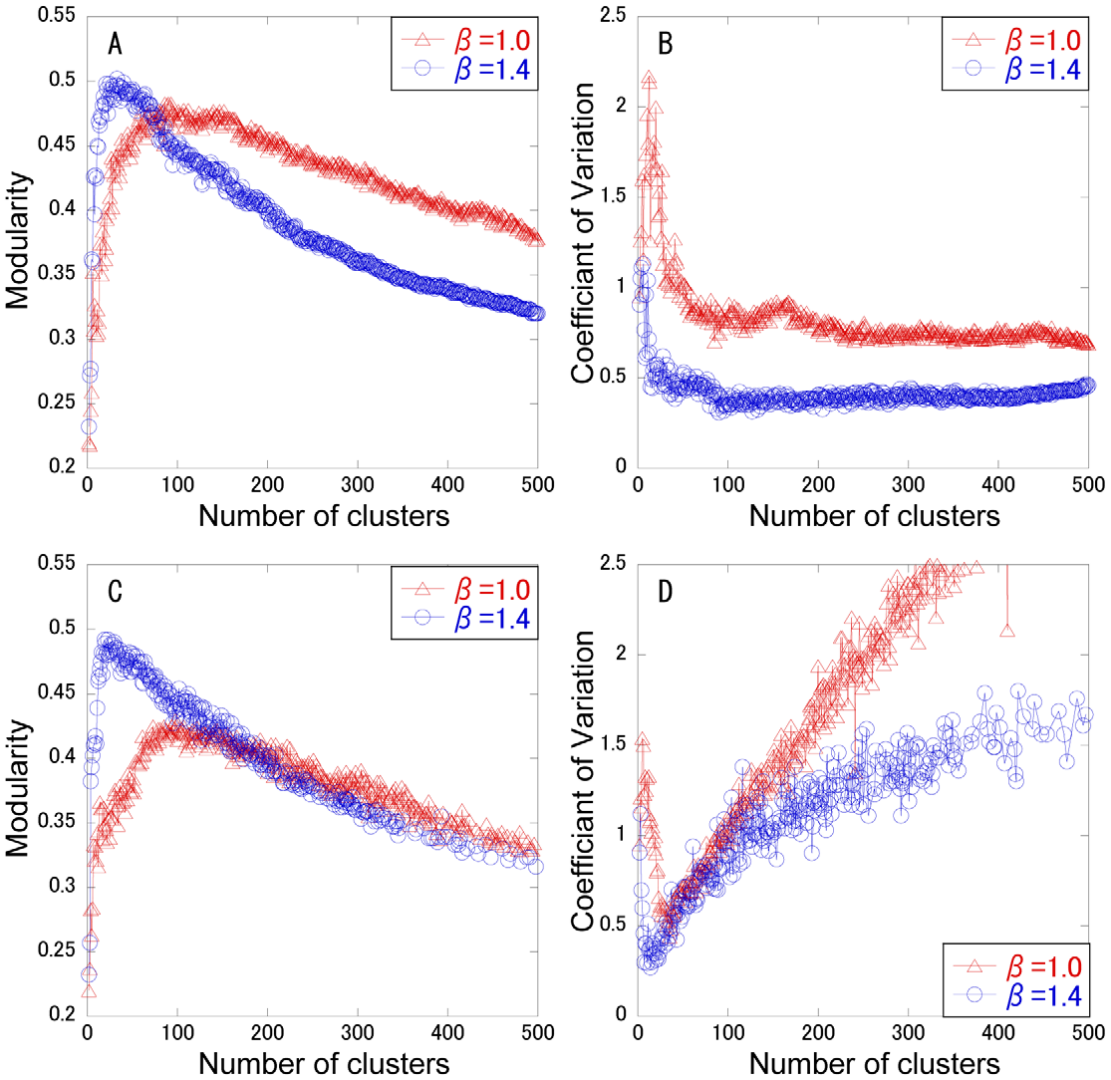
Changes in modularity and CV of cluster size with respect to cluster numbers. The β factor is set to 1 (triangle) or 1.4 (circle) for ADMSC. The modularity (A, C) and the CV of cluster size (B, D) are calculated at each cluster number. The complete linkage method (A, B) and k-means method (C, D) are performed in hierarchical and non-hierarchical clustering analyses, respectively.

In spectral analysis, the change curve of eigenvalues can generally be a measure to estimate the number of clusters. In scale free or heterogeneous networks, however, it is often hard to identify the cluster number, because there is no great gap between the neighboring eigenvalues (Figure S2). Thus, the cluster number is determined that maximizes the modularity. Modularity maximization is one of the most widely used methods for community or cluster detection. The modularity and the CV for cluster size are calculated with respect to the cluster number, as shown in Figure 1. As a control, the normal spectral analysis (β = 1) is used. Two typical clustering methods: k-means and completed linkage method are employed to divide the (*k*-1) dimensional diffusion map into *k* clusters. The modularity shows a convex curve with the maximum value. In the complete linkage method, the modularity for β = 1.4 is higher than that for β = 1 below a cluster number of 90 (Figure 1A), although the modularity for β = 1 is higher than that for β = 1.4 above it. ADMSC with β = 1.4 provides the highest modularity of 0.502 at a cluster number of 33. In the k-means method, ADMSC with β = 1.4 shows higher modularity than that with β = 1 below a cluster number of 99, providing the highest modularity (0.492) (Figure 1C). As far as the CV of cluster size is concerned, in both the methods the CVs of cluster size for β = 1.4 are smaller than those for β = 1. In the complete linkage method, the CVs for β = 1.4 are greatly suppressed less than 0.6 above a cluster number of 14, whereas they are not so greatly in the k-means method. This shows that the complete linkage method can generate the clusters with a less variation in size. In addition, the calculation speed for the complete linkage method is 3-fold enhanced than that for the k-means method (data not shown). Since the complete linkage method provides higher modularity, higher speed, and less CVs of cluster size than the k-means method, the complete linkage method is selected for clustering the diffusion map of the PPI network. The cluster number is determined as 33, the number that maximizes modularity.

**Figure 2:**
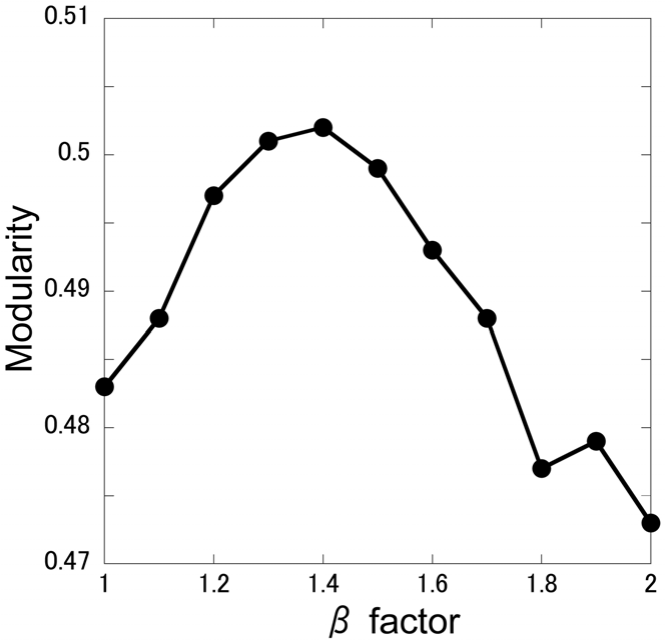
Effect of the β factor on modularity.

Next, to demonstrate how a β factor of 1.4 is selected, the effects of β on the modularity are illustrated in Figure 2, where the cluster number that maximizes the modularity at each β is plotted with respect to β. A β factor of 1.4 is shown to maximize the modularity, which is a reasonable choice for ADMSC.

### Characterization of clustering performance in comparison with other methods

To further demonstrate the feasibility of ADMSC, we compared its performance with established methods: MCL and SPB, as shown in Table 1. The cluster number for MCL is uniquely determined as 1233. The cluster number for SPB is set to 319 that maximizes the modularity. In addition, SPB is performed at a cluster number of 33, the optimal number for ADMSC. The cluster number of ADMSC is set to 33 (optimal number), 319, and 1233, so that ADMSC can be compared with SPB and MCL at the same cluster numbers.

**Table 1:** Characterization of the performance of ADMSC.

Method	Time	Modularity	Number of clusters	CV of cluster size
ADMSC				
(β = 1.0)	125 (sec)	0.436	33	0.83
(β = 1.4)	125 (sec)	0.502	33	0.48
(β = 1.4)	204 (sec)	0.355	319	0.41
(β = 1.4)	1071(sec)	0.227	1233	0.62*^1^
SPB	23(hour)	0.239	33	4.56
SPB	229(hour)	0.506	319	2.49*^2^
MCL	1750(sec)	0.275	1233	1.22*^3^

The inflation parameter of MCL is set to 2. The data *1, *2, and *3 contain 49, 20 and 152 singletons, respectively. The simulations are carried out by CPU Core 2 Duo E6850 (3GHz) with Memory 3.25 Gbyte.

First, the calculation rates are characterized. SPB needs longer time than any other methods. SPB is not readily applicable to a large-scale network with thousands of nodes. ADMSC shows the highest speed at a cluster number of 33, while the calculation time required by ADMSC increases with an increase in the cluster number. The difference in the calculation speed of ADMSC is caused not by the spectral analysis but by the complete linkage method. ADMSC is the fastest algorithm for network clustering in this study.

**Figure 3:**
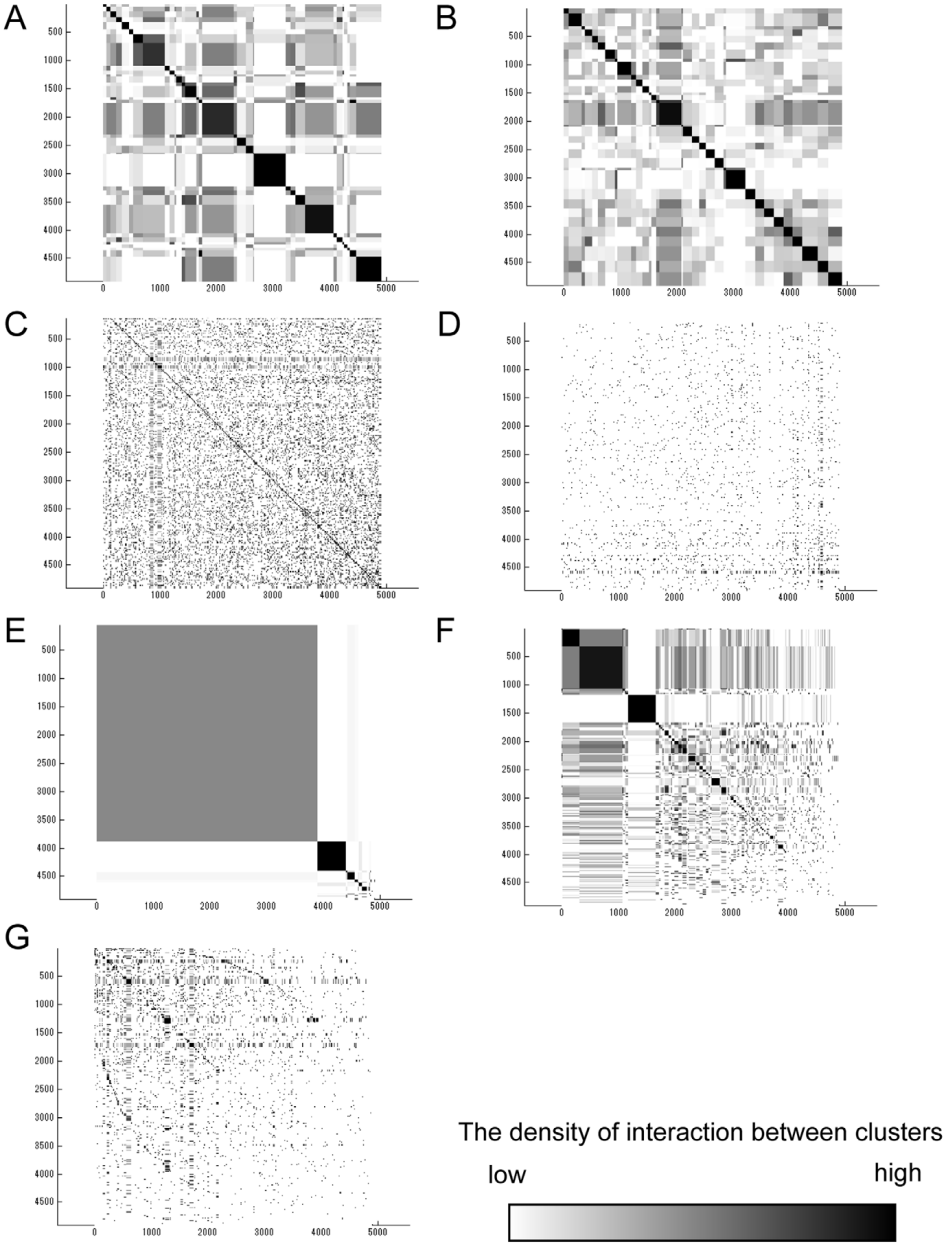
Distributions of cluster size in a yeast PPI network. Proteins are arranged in both the vertical and horizontal axes in the same turn. The density of interactions between clusters is marked as the degree of darkness. The size of the squares on the diagonal indicates the cluster size, and the darkness density shows the interaction strength of the inter- and intra-clusters. The cluster size distributions are calculated by: (A) ADMSC with β = 1 (regular spectral analysis) at a cluster number of 33, (B) ADMSC with β = 1.4 at a cluster number of 33, (C) ADMSC with β = 1.4 at a cluster number of 319, (D) ADMSC with β = 1.4 at a cluster number of 1233, (E) SPB at a cluster number of 33, (F) SPB at a cluster number of 319, and (G) MCL at a cluster number of 1233.

Second, the CVs of cluster size for ADMSC are less than 1 at any cluster number and smaller than those for SPB and MCL (4.56, 2.49 and 1.22), indicating that ADMSC presents the clusters with approximately same sizes. The distributions for cluster sizes are illustrated in Figure 3. ADMSC with β = 1.4 partitions the network approximately equally (Figure 3BCD). SPB provides a few large clusters with many tiny ones (Figure 3EF). MCL produces 1233 fine clusters (Figure 3G), which is due to the fact that MCL is originally designed for detecting protein complexes.

Third, ADMSC with β = 1.4 shows a high modularity of 0.502 at a cluster number of 33. The modularity calculated by ADMSC decreases with an increase in the cluster number (33, 319, 1233). SPB provides a high modularity of 0.506 at 319, although it generates a few giant cluster with many tiny ones (cluster size = 1) (Table 1). The modularity of ADMSC (33 clusters) is comparable to that of SPB (319 clusters). The modularity by MCL is rather low, confirming that MCL is not designed for network partition but for finding protein complexes.

**Figure 4:**
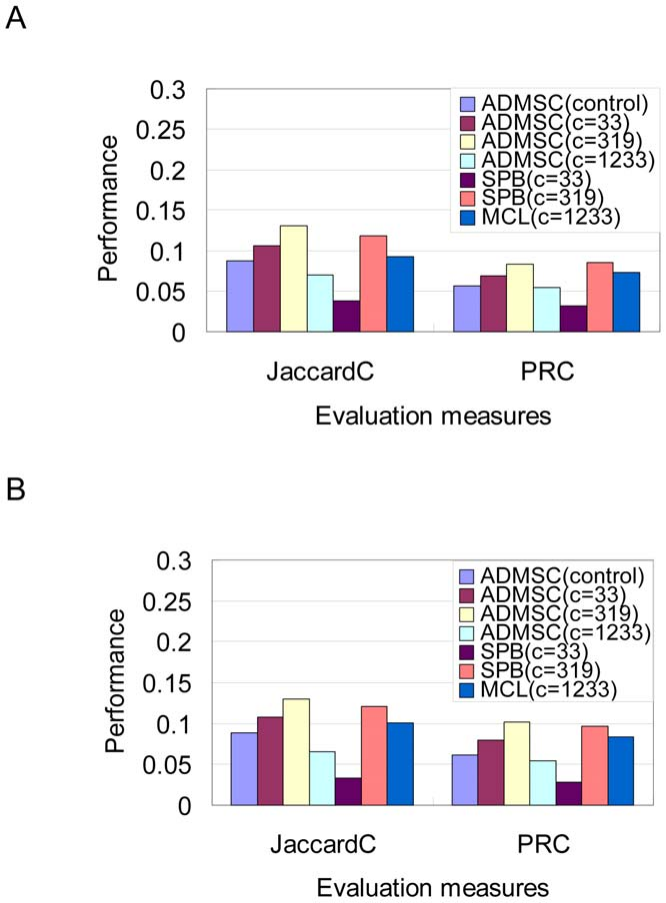
Clustering performance judged by JaccardC and PRC regarding BP and CC groups. (A) Biological Process groups. (B) Cellular Component groups. “c” indicates the number of clusters. ADMSC (control): β is set to one, where the number of clusters is 33 (c = 33). ADMSC (c = 33): β is set to 1.4.

Finally, to demonstrate how the clusters estimated by ADMSC overlap the known groups of proteins, we compare the overlap measures between ADMSC and established methods: MCL and SPB. The JaccardC and PRC regarding BP and CC groups are shown in Figure 4. While the perfect consistency between the topological and biological clusters is not theoretically guaranteed, it is known that there are correlations between them from experiences.

The introduction of β = 1.4 increases the JaccardC and PRC values regarding both the BP and CC groups, compared with the normal spectral clustering (β = 1), demonstrating that the adjustable parameter is effective in enhancing the clustering performance. While ADMSC with the optimal cluster number (33) shows relatively high values of JaccardC and PRC, it presents a little bit smaller values of them than SPB with the optimal cluster number (319). The ADMSC-estimated cluster structure may not be so highly correlated to the GO-based biological modules as the SPB-estimated one. When the same cluster number (33) is employed, ADMSC provides higher values of modularity, JaccardC and PRC than SPB. At a cluster number of 319, ADMSC also shows higher JaccardC and PRC than SPB, although the modularity for ADMSC (0.355) is lower than that for SPB (0.506). It suggests that ADMSC has the potential to partition a network into biologically meaningful modular structures and the modularity (a topological measure) does not necessarily reflect GO-based biological modules. Determination of the cluster number is suggested to affect the clustering performance.

Compared with MCL, ADMSC with the optimal cluster number (33) provides comparable or high values of JaccardC and PRC. The ADMSC-estimated cluster structure can be more correlated to the GO-based biological modules than the MCL-estimated one. On the other hand, when the same cluster number (1233) is employed, the values of modularity, JaccardC and PRC for ADMSC are all less than those for MCL. This is because ADMSC is not designed to find clusters with small size or protein complexes, differing from MCL.

In summary ADMSC takes an advantage in the fast partition of PPI networks into biologically significant clusters with approximately equal sizes. ADMSC is not applicable to finding protein complexes, but to clear partition of large-scale networks.

### Robustness analysis

**Figure 5:**
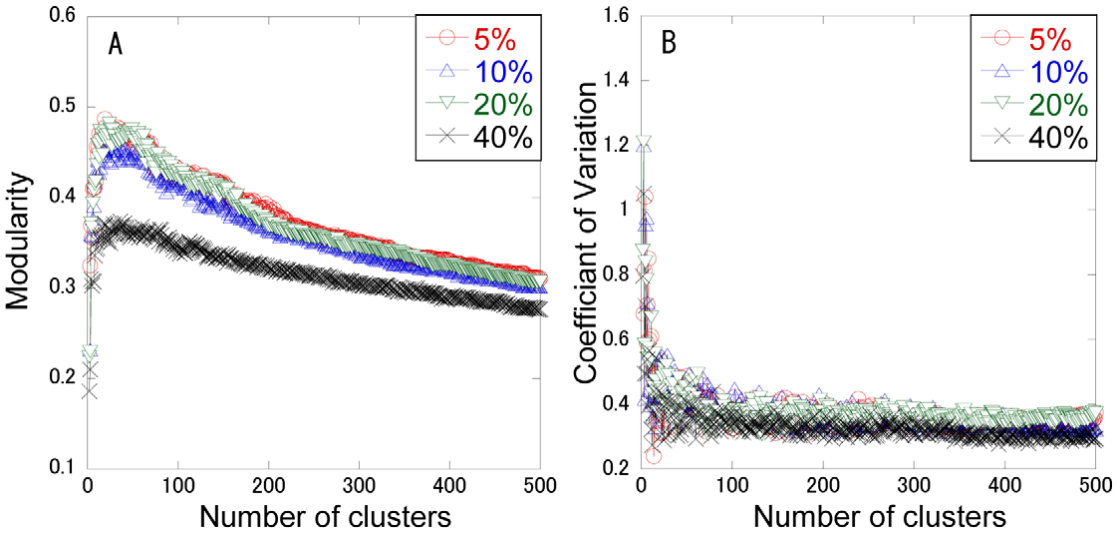
Perturbation analysis for modularity and cluster size calculated by ADMSC. Changes in the modularity (A) and the CV of cluster size (B) are calculated by ADMSC with respect to perturbed edges in the yeast PPI network. Five, ten, twenty, and forty percentages of edges are replaced randomly.

**Figure 6:**
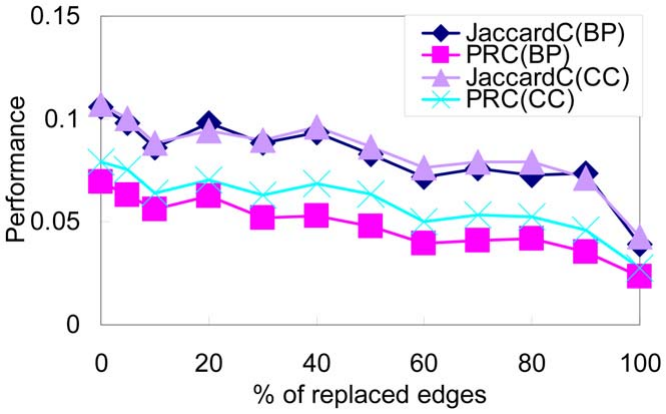
Perturbation analysis of JaccardC and PRC for the clusters estimated by ADMSC. 5–100 percentages of edges are replaced randomly.

To characterize the robustness of ADMSC, we randomly replace 5 to 100 percentages of edges and investigate the change in modularity, the CV of cluster size and the cluster mapping measures (JaccardC and PRC), as shown in Figures 5 and 6. The modularity is not greatly varied with respect to 5 to 20 percentages of the perturbed edges. The CV of cluster size is not greatly changed for 5 to 40 percentages of them. The JaccardC and PRC are robust with respect to 5 to 20 percentages of perturbed edges. Theses results demonstrate that ADMSC is rather robust with respect to perturbations, i.e., experimental errors.

### Application to other PPI networks

**Figure 7:**
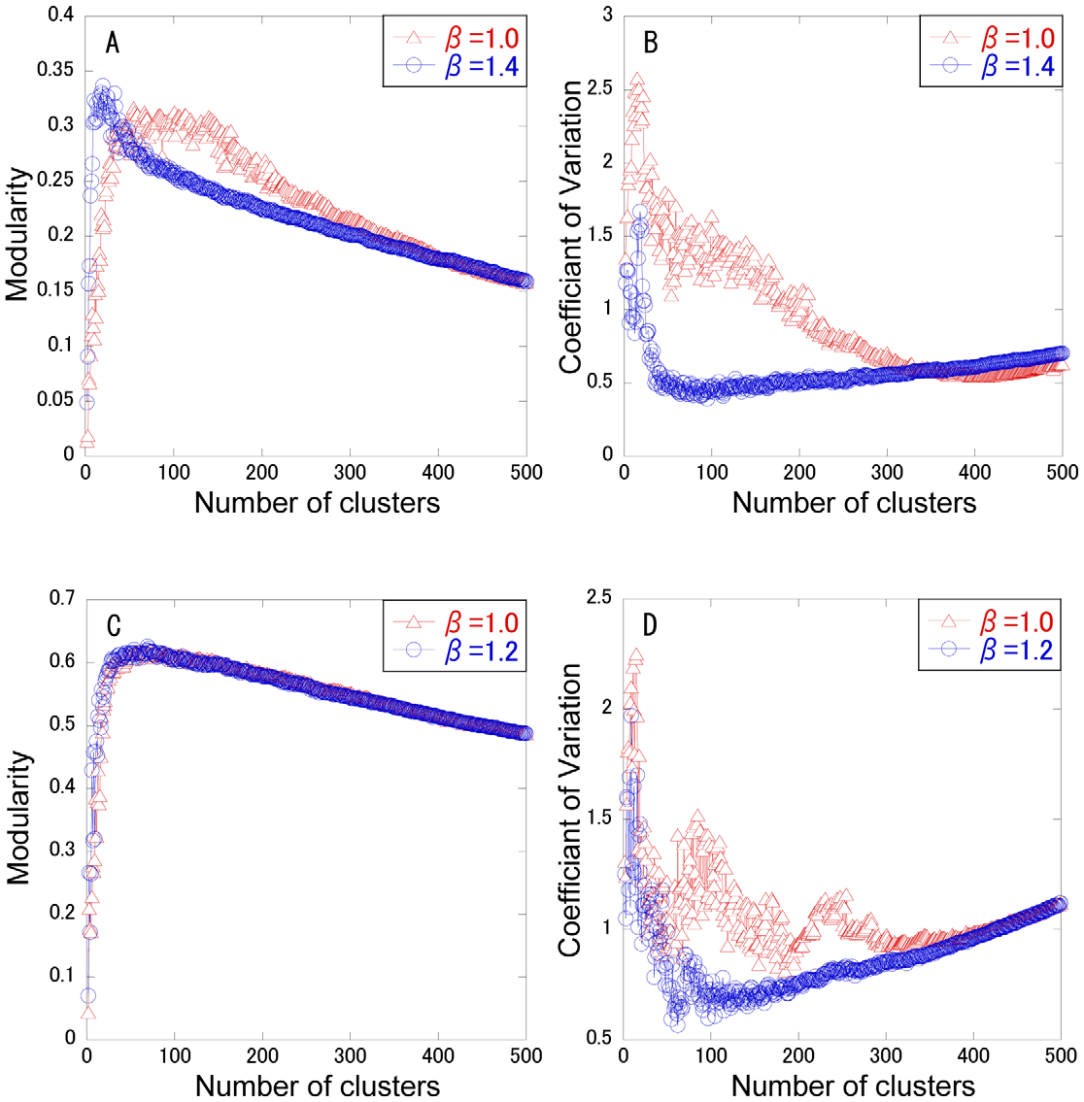
Application of ADMSC to other PPI networks. The modularity (A) and the CVs of cluster size (B) are plotted with respect to cluster number in *Escherichia coli*. The modularity (C) and the CV of cluster size (D) are plotted with respect to cluster number in *Caenorhabditis elegans*.

Finally, to demonstrate the applicability of ADMSC, it is applied to the PPI networks of *E. coli* and *C. elegans*, as shown in Figure 7. The modularity and CVs of cluster size are plotted with respect to the cluster number. In *E. coli*, ADMSC with β = 1.4 presents a higher modularity than that with β = 1.0 below a cluster number of less than 55, while it becomes less than that with β = 1.0 above it. ADMSC with β = 1.4 provides the highest modularity at a cluster number of 20. The CV of cluster size for β = 1.4 is suppressed in comparison with that for β = 1.0. In *C. elegans*, ADMSC with β = 1.2 provides a higher modularity than ADMSC with β = 1.0 below a cluster number 65, indicating the highest modularity at a cluster number of 69. The CV of cluster size for β = 1.2 is smaller than that for β = 1.0. Use of the β factor takes an advantage in obtaining the highest modularity and in identifying the clusters with a small variation in size.

## Discussion

It is hard for traditional clustering methods, which employ similarity measures between a pair of nodes to perform agglomerative approaches, to partition heterogeneous PPI networks into clusters with approximately equal sizes. In this background, we get insight into the spectral clustering approach so that it can be available to PPI networks. The clear decomposition by ADMSC could be attributed to two factors: the selection of the angular distance between nodes in the diffusion map and the introduction of the β factor to adjust the spectrums of eigenvectors to the heterogeneity of the networks.

### Angular distance

Generally, the lengths between a pair of nodes are employed as similarity measures in ordinary clustering. However, use of these measures leads to identification non-separable giant clusters in PPI networks. On the other hand, spectral clustering transforms binary data into the multi-dimensional Euclidean space spanned by eigenvectors to illustrate the geometrical (diffusion) map of all the nodes, thereby enabling use of various distances. A breakthrough of ADMSC is the use of angular distance that is calculated from the geometrical map (coordinates) of all the nodes. In the diffusion map, the nodes seem to exist in a uniform distribution within a ball, but they are actually distributed along the radial directions from the original point, forming cluster structure. Such node distributions can be confirmed by visualizing three-dimensional diffusion maps of test network models (Figure S3). Therefore, use of angular distance can divide the network nodes into clusters with a small variation in size. An understanding of the node distribution in the diffusion map is critically important for ADMSC.

### Power of the β factor

The β factor can be regarded as the unique factor that weights the network topology according to the node degree. For β = 0 or 1, ADMSC coincides with the cases considered in the spectral analysis for the usual or normalized graph Laplacian, respectively (Table S1). For homogenous networks (random or regular networks) the diffusion models are insensitive to a change in β because all nodes have roughly equal degrees. On the other hand, for highly heterogeneous PPI networks, transformation with the β factor is effective in adjusting the clustering approach to the heterogeneity of the networks.

To determine the exact value of β, it is recommended to systematically search the two parameters of the β factor and cluster number that provide the highest modularity. The maximization of modularity is one of widely-used criteria and can be reformulated as an eigenvector problem of the spectral clustering algorithm [33], [41]. Since ADMSC is a fast and robust algorithm, such a systematic search is an easy task. Based on the simulations, a suitable value of β can be from 1 to 2.

### Fast, robust, simple algorithm

Here, ADMSC can be compared with the ensemble method, because the ensemble method is supposed to show high clustering performance for network partition [10], but it is not used in our quantitative comparison due to complexity of its procedures. The ensemble method consists of multiple procedures: weighting an adjacency matrix, applying multiple base partitioning algorithms to the weighted matrix, and performing principal component analysis to find the consensus clusters calculated by the base clustering methods, thereby enabling finding biologically-significant clusters. Although we do not directly compare the clustering performance and modularity between them, ADMSC would take advantages in a fast and simple method without multiple redundant clustering nor consensus identification.

In conclusion, ADMSC presents the fast partition of large-scale PPI networks into biological clusters with approximately equal sizes, while it is very robust and appealing simple.

## Supporting Information

Figure S1 Degree distribution in the scale-free network of a yeast PPI network.
(0.05 MB PDF)

Figure S2 Changes in eigenvalues with respect to cluster number in the yeast PPI network.
(0.06 MB PDF)

Figure S3 Diffusion maps for a scale-free network and a network with distinct clusters.
(0.08 MB PDF)

Table S1 Relation to spectral analysis.
(0.09 MB PDF)

Table S2 Instruction of ADMSC program in Matlab.
(0.06 MB PDF)

## References

[1] Barabasi AL, Oltvai ZN (2004). Network biology: understanding the cell's functional organization.. Nat Rev Genet.

[2] Arnau V, Mars S, Marin I (2005). Iterative cluster analysis of protein interaction data.. Bioinformatics.

[3] Rives AW, Galitski T (2003). Modular organization of cellular networks.. Proc Natl Acad Sci U S A.

[4] Friedel CC, Zimmer R (2006). Inferring topology from clustering coefficients in protein-protein interaction networks.. BMC Bioinformatics.

[5] Pereira-Leal JB, Enright AJ, Ouzounis CA (2004). Detection of functional modules from protein interaction networks.. Proteins.

[6] Dunn R, Dudbridge F, Sanderson CM (2005). The use of edge-betweenness clustering to investigate biological function in protein interaction networks.. BMC Bioinformatics.

[7] Luo F, Yang Y, Chen CF, Chang R, Zhou J (2007). Modular organization of protein interaction networks.. Bioinformatics.

[8] Newman ME (2004). Fast algorithm for detecting community structure in networks.. Phys Rev E Stat Nonlin Soft Matter Phys.

[9] Newman ME, Girvan M (2004). Finding and evaluating community structure in networks.. Phys Rev E Stat Nonlin Soft Matter Phys.

[10] Asur S, Ucar D, Parthasarathy S (2007). An ensemble framework for clustering protein-protein interaction networks.. Bioinformatics.

[11] Bader GD, Hogue CW (2003). An automated method for finding molecular complexes in large protein interaction networks.. BMC Bioinformatics.

[12] King AD, Przulj N, Jurisica I (2004). Protein complex prediction via cost-based clustering.. Bioinformatics.

[13] Bu D, Zhao Y, Cai L, Xue H, Zhu X (2003). Topological structure analysis of the protein-protein interaction network in budding yeast.. Nucleic Acids Res.

[14] Sen TZ, Kloczkowski A, Jernigan RL (2006). Functional clustering of yeast proteins from the protein-protein interaction network.. BMC Bioinformatics.

[15] Van Dongen S (2000). Graph clustering by flow simulation [PhD thesis].

[16] Blatt M, Wiseman S, Domany E (1996). Superparamagnetic clustering of data.. Phys Rev Lett.

[17] Gagneur J, Krause R, Bouwmeester T, Casari G (2004). Modular decomposition of protein-protein interaction networks.. Genome Biol.

[18] Morrison JL, Breitling R, Higham DJ, Gilbert DR (2006). A lock-and-key model for protein-protein interactions.. Bioinformatics.

[19] Andreopoulos B, An A, Wang X, Faloutsos M, Schroeder M (2007). Clustering by common friends finds locally significant proteins mediating modules.. Bioinformatics.

[20] Royer L, Reimann M, Andreopoulos B, Schroeder M (2008). Unraveling protein networks with power graph analysis.. PLoS Comput Biol.

[21] Belkin M, Niyogi P (2003). Laplacian Eigenmaps for Dimensionality Reduction and Data Representation.. Neural Computation.

[22] Fischer I, Poland J (2004). New methods for spectral clustering..

[23] Meila M, Shi J, Lee TK, Dietterich TG, Tresp V (2001). Learning segmentation by random walks.. Advances in Nerual Information Processing Systems.

[24] Ng A, Jordan M, Weiss Y (2001). On spectral clustering: Analysis and an algorithm.

[25] Spirin V, Mirny LA (2003). Protein complexes and functional modules in molecular networks.. Proc Natl Acad Sci U S A.

[26] Yoon J, Blumer A, Lee K (2006). An algorithm for modularity analysis of directed and weighted biological networks based on edge-betweenness centrality.. Bioinformatics.

[27] Nadler B, Lafon S, Coifman RR, Kevrekidis IG (2005). Diffusion Maps, Spectral Clustering and Eigenfunctions of Fokker-Planck operators..

[28] Yen L, Vanvyve D, Wouters F, Fouss F, Verleysen M (2005). Clustering using a random-walk based distance measure.. ESANN.

[29] Eriksen KA, Simonsen I, Maslov S, Sneppen K (2003). Modularity and extreme edges of the internet.. Phys Rev Lett.

[30] Kozma B, Hastings MB, Korniss G (2005). Diffusion processes on power-law small-world networks.. Phys Rev Lett.

[31] Koren Y (2003). On spectral graph drawing.. Lect Notes Comput Sci.

[32] Donetti L, Munoz M (2004). Detecting network communities: a new systematic and efficient algorithm.. J Stat Mech.

[33] Newman ME (2006). Modularity and community structure in networks.. Proc Natl Acad Sci U S A.

[34] Boyle EI, Weng S, Gollub J, Jin H, Botstein D (2004). GO::TermFinder–open source software for accessing Gene Ontology information and finding significantly enriched Gene Ontology terms associated with a list of genes.. Bioinformatics.

[35] Song J, Singh M (2009). How and when should interactome-derived clusters be used to predict functional modules and protein function?. Bioinformatics.

[36] Brohee S, van Helden J (2006). Evaluation of clustering algorithms for protein-protein interaction networks.. BMC Bioinformatics.

[37] Salwinski L, Miller CS, Smith AJ, Pettit FK, Bowie JU (2004). The Database of Interacting Proteins: 2004 update.. Nucleic Acids Res.

[38] Kurata H, Matoba N, Shimizu N (2003). CADLIVE for constructing a large-scale biochemical network based on a simulation-directed notation and its application to yeast cell cycle.. Nucleic Acids Res.

[39] Li W, Kurata H (2005). A grid layout algorithm for automatic drawing of biochemical networks.. Bioinformatics.

[40] Li W, Kurata H (2008). Visualizing Global Properties of Large Complex Networks.. PLoS One.

[41] Jiang J, Dress A, Yang G (2009). A spectral clustering-based framework for detecting community structures in complex networks.. Applied Mathematics Letters.

