# Supplementary material for: Diffusion Model Based Spectral Clustering for Protein-Protein Interaction Networks
Source: PLoS One. 2010 Sep 7;5(9):e12623. doi: 10.1371/journal.pone.0012623 (PMC2935381; doi:10.1371/journal.pone.0012623)
Supplement: Figure S2 — Changes in eigenvalues with respect to cluster number in the yeast PPI network. (0.06 MB PDF) [file pone.0012623.s002.pdf]

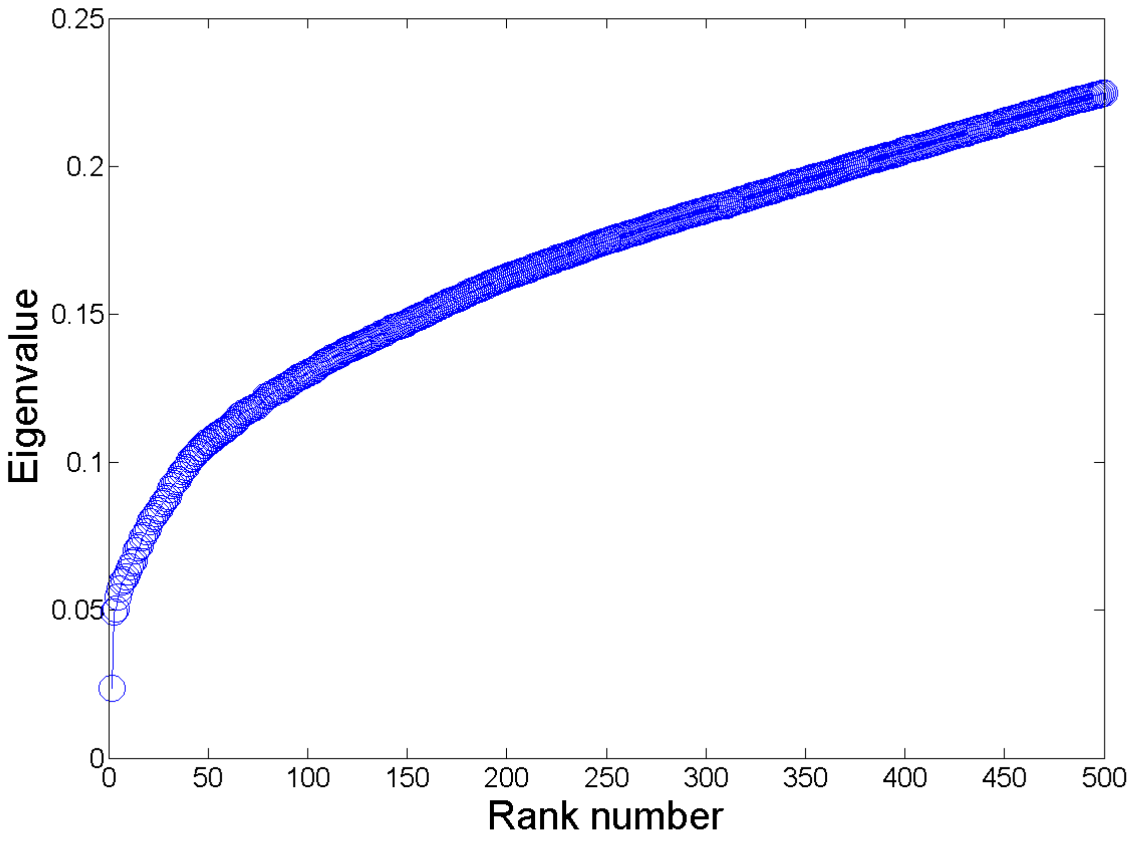

Figure S2 Changes in eigenvalues with respect to cluster number in the yeast PPI network

The eigenvalues for the diffusion matrix are sorted in the ascending order. There is no great gap between the neighboring eigenvalues.
