# Supplementary material for: Diffusion Model Based Spectral Clustering for Protein-Protein Interaction Networks
Source: PLoS One. 2010 Sep 7;5(9):e12623. doi: 10.1371/journal.pone.0012623 (PMC2935381; doi:10.1371/journal.pone.0012623)
Supplement: Figure S3 — Diffusion maps for a scale-free network and a network with distinct clusters. (0.08 MB PDF) [file pone.0012623.s003.pdf]

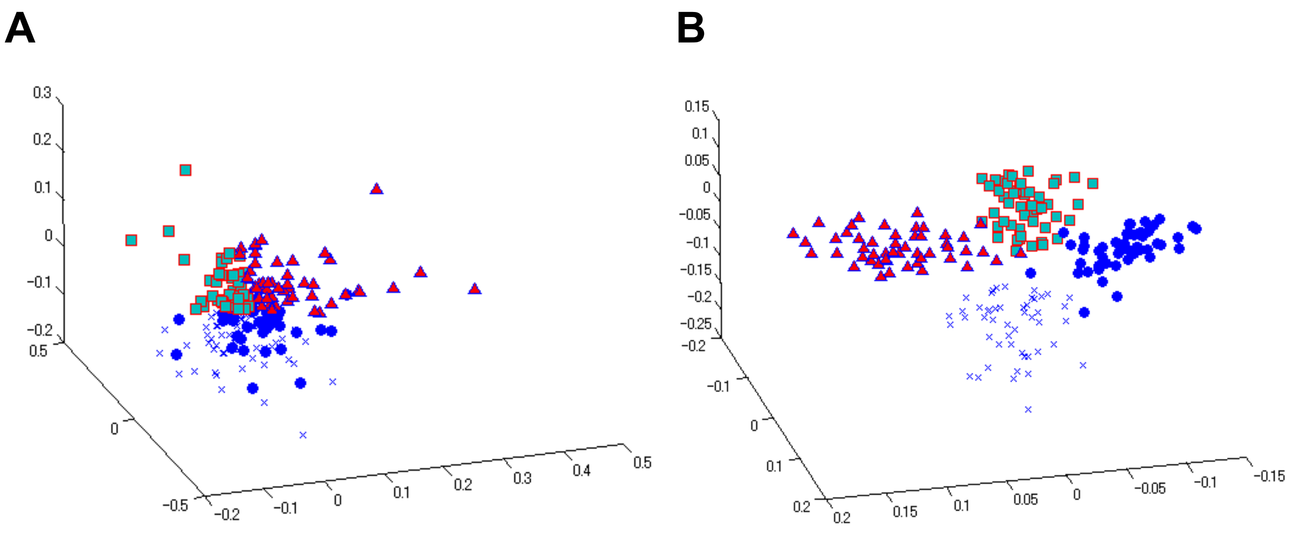

Figure S3 Diffusion maps for a scale free network and a network with distinct clusters.

The diffusion map spanned by three eigenvector is shown in three-dimensional space. The same symbol belongs to the same cluster.

(A) A scale free network (200 nodes and 398 edges) is built based on the BA (Barabási and Albert) model and decomposed into four groups by ADMSC.

(B) An artificial network with four distinct clusters (200 nodes and 994 edges) is built and decomposed into four groups by ADMSC.

In (B), the clear clusters are illustrated along the radial directions from the original point. In (A), while the nodes are condensed around the original point, each cluster seems to exist along the radial directions from the original point. It suggests that use of the angular distance partitions a scale free network.

To clearly demonstrate the cluster structure of these networks, we provide DiffMap_BAmodel.fig (A) and DiffMap_FourCluster.fig (B) in ADMSC.zip. These figures can be rotated in MATLAB.
