# Supplementary material for: Diffusion Model Based Spectral Clustering for Protein-Protein Interaction Networks
Source: PLoS One. 2010 Sep 7;5(9):e12623. doi: 10.1371/journal.pone.0012623 (PMC2935381; doi:10.1371/journal.pone.0012623)
Supplement: Table S1 — Relation to spectral analysis. (0.09 MB PDF) [file pone.0012623.s004.pdf]

Table S1 Relation to spectral analysis

Generally, the Laplacian matrix of a graph network is defined as:

$$\mathbf{L} = \mathbf{\Delta} - \mathbf{A} \tag{S1}$$

where $\mathbf{A}$ is the adjacency matrix. $\mathbf{\Delta}$ is given by:

$$\Delta_{ij} = \delta_{ij} \sum_{k=1}^{n} A_{ik} \qquad \delta_{ij} = \begin{cases} 1 \; if \;\; i = j \\ 0 \; if \;\; i \neq j \end{cases} \tag{S2}$$

When use of the transition matrix of a random walk $\mathbf{D}^{-1}\mathbf{A}$ is favored, the Laplacian matrix is changed into:

$$\mathbf{L}^{*} = \mathbf{\Delta}^{*} - \mathbf{D}^{-1}\mathbf{A} \tag{S3}$$

$$\Delta^{*}_{ij} = \delta_{ij} \sum_{k=1}^{n} (\mathbf{D}^{-1}\mathbf{A})_{ik} \tag{S4}$$

$$\delta_{ij} = \begin{cases} 1 \; if \;\; i = j \\ 0 \; if \;\; i \neq j \end{cases}$$

Here, the set of all the eigenvalues λ and the corresponding eigenvectors $\mathbf{v}$ for $\mathbf{L}^{*}$, called spectrum, is given by:

$$\mathbf{L}^{*} \cdot \mathbf{v}_i = \lambda \cdot \mathbf{v}_i \tag{S5}$$

where $\{\mathbf{v}_i\}$ provides the node coordinates in the Euclidean space to perform network clustering. Since ADMSC corresponds to the spectral clustering that employs a modified transition matrix $\mathbf{D}^{-\beta}\mathbf{A}$, it can be regarded as the diffusion model-based probabilistic interpretation of spectral analysis.
