# Supplementary material for: Diffusion Model Based Spectral Clustering for Protein-Protein Interaction Networks
Source: PLoS One. 2010 Sep 7;5(9):e12623. doi: 10.1371/journal.pone.0012623 (PMC2935381; doi:10.1371/journal.pone.0012623)
Supplement: Table S2 — Instruction of ADMSC program in Matlab. (0.06 MB PDF) [file pone.0012623.s005.pdf]

Table S2 Instruction of ADMSC program in Matlab

README.txt in ADMSC.zip
Execute the ADMSC.m file in MATLAB<br>>>ADMSC<br><br><br>Input for ADMSC.m<br>line 46: nc=33; % Number of clusters<br>line 47: B=1.4; % Beta factor<br>*You decide the number of clusters and the value of the beta factor.<br><br>line 52 : [nodea nodeb] = textread('Scere.txt','%s%s'); % input your network<br>*The file of default is the yeast PPI network that consists of the binary relation between proteins.<br><br>line 97 : outputdata(nodes,cidx,modu,cv,nnode,nedge); % output data<br>*The result is output as ‘output.txt’.
