# Supplementary material for: Diffusion Model Based Spectral Clustering for Protein-Protein Interaction Networks
Source: PLoS One. 2010 Sep 7;5(9):e12623. doi: 10.1371/journal.pone.0012623 (PMC2935381; doi:10.1371/journal.pone.0012623)
Supplement: Figure S1 — Degree distribution in the scale-free network of a yeast PPI network. (0.05 MB PDF) [file pone.0012623.s001.pdf]

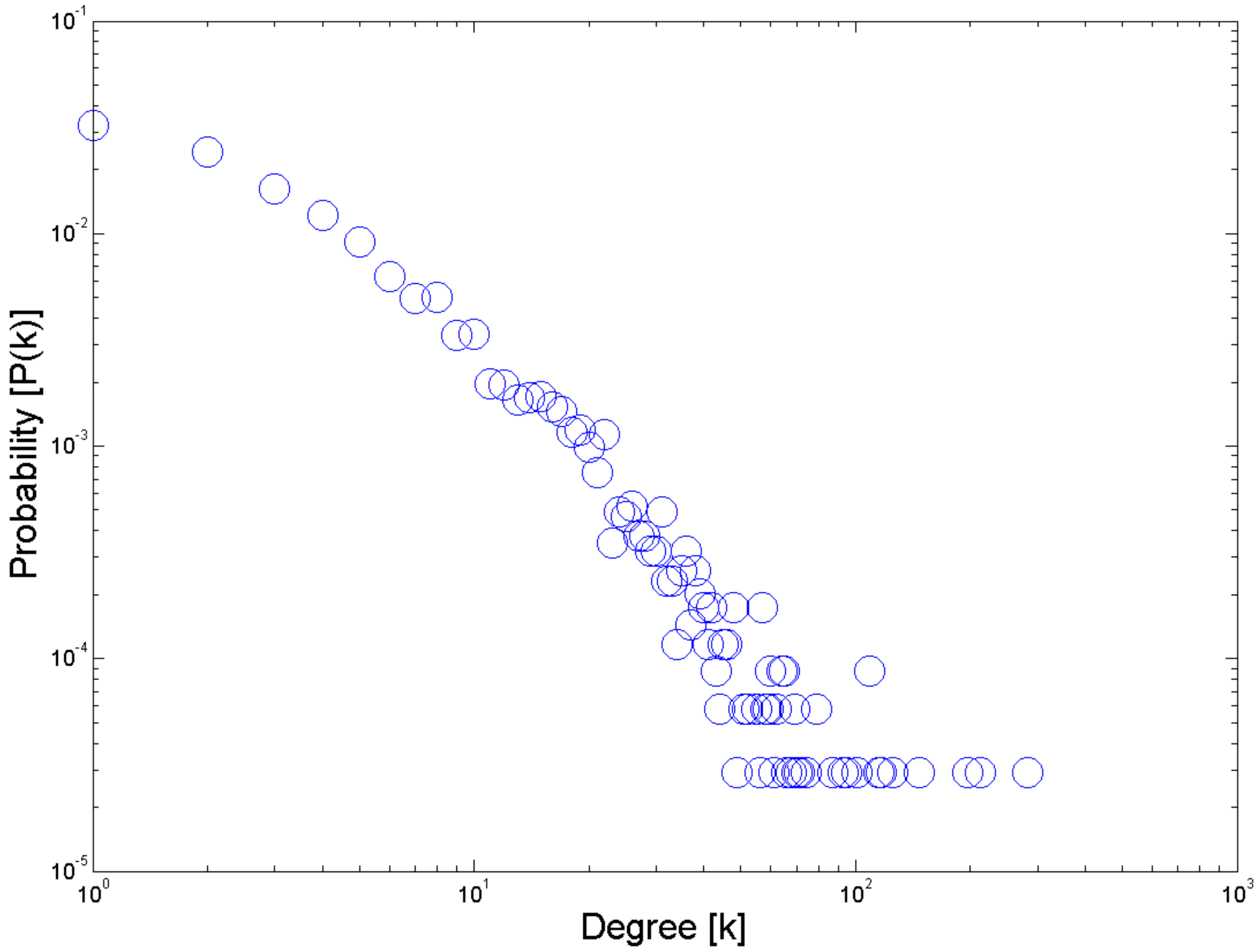

Figure S1 Degree distribution in the scale free network of an yeast PPI network

k is the node degree.
